# Summary of the proceedings of the International Forum 2020: “Radiologists fighting COVID-19: a united response to a global crisis”

**DOI:** 10.1186/s13244-020-00959-3

**Published:** 2021-02-17

**Authors:** European Society of Radiology (ESR)

**Affiliations:** European Society of Radiology (ESR), Am Gestade 1, 1010 Vienna, Austria

**Keywords:** COVID-19, Guidelines, Measures, Strategies

## Abstract

The ESR International Forum at the ECR 2020 discussed the current situation, activities and measures undertaken by radiologists around the world in their fight against the global COVID-19 pandemic. The participating societies were invited to submit written reports detailing the current situation in their countries or regions. The European Society of Radiology (ESR) established the ESR International Forum in order to discuss hot topics in the profession of radiology with non-European radiological partner societies. At the ESR International Forum 2020, different strategies, initiatives, and ideas were presented with regard to facing this unprecedented challenge together.

## Main messages

- It is important to understand the main characteristics of the novel COVID-19 virus and radiologists play a crucial role in that regard.
- Radiological societies around the world are creating guidelines and recommendations based on the latest findings by radiologists fighting on the front line.
- Ensuring the safety and well-being of the patients as well as the entire medical staff when dealing with the virus is of paramount importance.
- Even though the current global focus of radiology is on COVID-19, other radiological examinations must not be ignored.
- Despite the restrictions imposed by countries in order to fight the virus, the radiological societies, now more than ever, need to work together and commit to sharing knowledge and experience.

## Introduction

The ESR International Forum was established by the with the aim of discussing pivotal themes in the field of radiology with international societies from outside Europe. The ESR International Forum is held every year at the European Congress of Radiology (ECR) and participation is by invitation only. Due to the unprecedent situation caused by the global pandemic of COVID-19, the ECR 2020 was held as a virtual congress and the participants of the ESR International Forum submitted written reports describing the steps and measures radiologists in their countries are undertraining in the fight against the global pandemic.

Previous topics discussed in the ESR International Forum include the relation between radiology and nuclear medicine, the position of ultrasound in radiology, the relation of general radiology and subspecialty radiology, the implementation of clinical decision support and imaging referral guidelines in the clinical routine, the position of interventional radiology within radiology, value-based radiology, and the strategies to engage the younger generation.

The following societies were invited to deliver a presentation and to present the point of view of their respective country or region: The American College of Radiology (ACR), the Asian Oceanian Society of Radiology (AOSR), the Brazilian College of Radiology (CBR), the Canadian Association of Radiologists (CAR), the Chinese Society of Radiology (CSR), the Colombian Association of Radiology (ACR), the Egyptian Society of Radiology and Nuclear Medicine (ESRNM), the Indian Radiological and Imaging Association (IRIA), the Inter-American College of Radiology (CIR), the International Society of Radiology (ISR), the Japan Radiological Society (JRS), the Korean Society of Radiology (KSR), the Mexican Federation of Radiology and Imaging (FMRI), the Mexican Society of Radiology and Imaging (SMRI), the Peruvian Society of Radiology (SOCPR), the Radiological Society of the Emirates (RSE), the Radiological Society of North America (RSNA), the Radiological Society of Thailand (RST), the Royal Australian and New Zealand College of Radiologists (RANZCR), the Royal College of Radiologists of Thailand (RCRT), and the Radiological and Diagnostic Imaging Society of Sao Paulo (SPR). The European Society of Radiology (ESR) presented the situation in Europe.

## The situation in North America

M. Barry submitted the report on behalf of the **Canadian Association of Radiologists (CAR)**. Pointing out the radiologists’ role in the diagnosis and assessment of COVID-19, but also in the resumption of radiology clinical services in a pandemic environment, M. Barry emphasized the close collaboration between the Canadian Association of Radiologist and its members and national organizations which resulted in the publishing of several guidelines and recommendation on the management of the COVID-19 and the resumption of radiology services. Additionally, many of CAR members have already published research articles and the CAR is receiving regular updates on the status of COVID-19 across the country, which should help increase the overall knowledge when dealing with this novel threat. The CAR also created a Task Force on the Resumption of Radiology Clinical Services and a report [1] with recommendations for safety protocols was published in May 2020 and has been enthusiastically received by the radiology community. A dedicated and daily updated COVID-19 resource page was created on the CAR website serving as a valuable reference for not only radiologists but for all Canadians regarding the impact of medical imaging in the management of COVID-19. Finally, M. Barry outlined the importance of advocacy and communication with the government, explaining that the CAR is in constant correspondence with relevant bodies and was invited to appear before the House of Commons Standing Committee on Health in June 2020, to stress the urgent need for medical image equipment, health human resources, as well as infrastructure support to help an already overburdened health system. In conclusion, M. Barry stated that the CAR is working with the government, industry, and stakeholders to provide information and advice which will minimize the spread of COVID-19 while safely moving forward with radiology clinical services.

J. P. Borgstede, reporting on behalf of the **Radiological Society of North America (RSNA)**, explained that, with a global membership, the RSNA is in a unique position to address global impact of COVID-19 and support radiologists at all stages of managing the pandemic. In that sense, the RSNA established two volunteer task forces to provide resources for the RSNA members during the outbreak. The RSNA COVID-19 Task Force has developed 4 recommendation documents, organized 6 free webinars and several videos to date and is planning sessions of COVID-19-related content for RSNA 2020. The RSNA AI COVID-19 Task Force has launched an initiative to create a global open data repository to support research on the use of medical imaging to help address the crisis. According to J. P. Borgstede, the RSNA has, to date, seen interest from over 200 institutions across 6 continents and a proposal was submitted to the National Cancer Institute to publish data acquired by this Task Force as a data collection on The Cancer Imaging Archive. A joint RSNA, AAPM (the American Association of Physicists in Medicine), and ACR whitepaper will be submitted to the National Institute of Health (NIH) and the National Institute of Biomedical Imaging and Bioengineering (NIBIB) with funding to support the repository initiative. Furthermore, an RSNA COVID-19 Community was established to help facilitate member discussion and peer-to-peer support around COVID-19-related topics and has, since its creation in April, grown to over 2,500 members. J. P. Borgstede also pointed out that the RSNA journals responded rapidly to the pandemic, soliciting guidance statements and establishing expedited peer review and publication processes. Since the first publication of images of the novel coronavirus in January, all five RSNA journals have published COVID-related content, with 93 articles in total. Finally, it was mentioned that these RSNA activities were covered by many major media outlets and that the RSNA received more than 4 million impressions from COVID-related stories on social media. In order to continue developing further steps, a survey was sent to RSNA members to assess what RSNA can do to help its members during the pandemic.

G. McGinty, on behalf of the **American College of Radiology (ACR),** pointed out that, as the “Voice of Radiology” with payers and policy makers, the American College of Radiology has issued guidance about the role of imaging and the need to prioritize safety by suspending non-urgent imaging. Given the initial shortages of Protective Personal Equipment (PPE), the ACR has also advocated for a safe working environment for the imaging community especially for technologists and trainees, many of whom were redeployed to “front line” care settings. A wholesale shift to remote working for many radiologists was supported by guidance from the ACR to inform safe and secure interpretations from home. Looking at the future consequences of the pandemic, G. McGinty explained that social distancing and cleaning requirements will make a return to prior volumes impractical for many and the financial future for some practices is uncertain. This will in turn have a knock-on effect on the job market for graduating trainees compounded by an anticipated recession and stock market volatility that will cause some senior radiologists to defer retirement. That is why the US Congress has authorized trillions of dollars in relief packages and, given that many radiologists in the US are independent practice owners, the ACR has provided invaluable guidance to members to help them navigate the complexities of qualifying for these programs. The ACR is also working actively with Congress to mitigate the impact of planned reimbursement cuts to radiology scheduled for 2021 with an average expected decrease in payment for imaging services of 10%. Commenting on the measures introduced by the Federal Government and many States, G. McGinty stated that the State of Emergency permitted relaxation of many regulations some of which are welcome and the ACR will lobby for their retention, while others, such as the expansion of scope of unsupervised practice of what are known as Advanced Practice Providers (APPs), will be threatening to radiologists in the longer term and the ACR will oppose their continuation. Commenting on the ACR Annual Meeting, it was stated that while the Meeting was converted to a virtual format it nevertheless succeeded in recreating the open debate and representative governance with even greater attendance participation at some sessions than normal. The ACR’s educational offerings, such as the American Institute of Radiologic Pathology, have also been converted to a virtual format with active work to ensure the use of appropriate tools and delivery to ensure a meaningful remote experience. The ACR’s Wellness initiative is actively working to support radiologists who may be working from home as well as juggling home schooling as well as dealing with existing burn out. Finally, G. McGinty explained that the ACR’s Center for Research and Innovation and its Data Science Institute are collaborating with other radiology and medical specialty societies to learn from the pandemic by establishing a registry of imaging and clinical data.

## The situation in Latin America

M. Hernandez Cruz, on behalf of the **Mexican Federation of Radiology and Imaging (FMRI),** reported on the current situation in Mexico. He pointed out that, as was the case in many other countries, Mexico’s health system was not prepared to face a contingency of such an unprecedented magnitude. Explaining that some guidelines were established and disseminated to the population to reduce infections, M. Hernandez Cruz pointed out that even with these measures, Mexico is experiencing high death tolls showing that the implemented strategies have not been entirely successful. He further explained that the Mexican government undertook a successful restructuring of the hospital system in which hundreds of public and private hospitals were modified and adapted to attend patients with COVID-19, thus successfully avoiding hospital saturation. However, as a result of insufficient protection materials, health personnel became infected, which in turn exacerbated the problem of the lack of medical staff and poor patient care. Health personnel, and in particular nurses and doctors, including radiologists, account for 20% of the total reported infected patients in Mexico. Because of this, radiology personnel have had to adapt to new conditions, especially methods of protection, such as the use of special clothing and masks. However, these actions are not always carried out due to the lack of protection supplies. Rotating medical and paramedical personnel, allowing less exposure to the virus, was another strategy taken to protect the staff. In order to keep radiologist updated, the Ministry of Health, Medical Societies, the private sector, and companies organized multiple online events for Mexican radiologists. Additionally, multiple international events from different radiological organizations, such as the European Society of Radiology, French Society of Radiology, Spanish Society of Radiology, Radiological Society of North America, and the Radiological Societies from several countries of Latin America were broadcasted and thus lead to expanding the knowledge and experience of Mexican radiologists. Besides this, teleradiology, the use of automated diagnostic systems, and the growth of artificial intelligence proved to be important tools supporting the role of diagnostic imaging. In conclusion, M. Hernandez Cruz stated that despite these significant advances in medical and technological knowledge, it cannot be denied that Mexico is a developing country and that its economic, technical, cultural, and governmental problems have contributed to this pandemic having even more significant consequences in Mexico than those observed in other countries around the world.

M. Palacios Montesinos on behalf of the **Mexican Society of Radiology and Imaging (SMRI)**, reported on the importance of continued medical education, explaining that the SMRI has conducted webinars on COVID-19 and has continued planning of the Annual Ultrasound Congress. However, at the same time, he emphasized the importance of resuming daily practices in the imaging area and establishing a series of rules and protocols that will allow safe procedures for both the radiologists and the technicians. To that end, various patient management protocols and the use of personal protective equipment, as well as deep cleaning of the rooms and medical equipment, were implemented. The use of personal protective equipment (PPE), including face masks, gowns, coveralls, gloves, goggles, and face shields remain essential. To further limit the risk of contagion, handwashing and the use of hand sanitizer in all service areas, as well as measuring the temperature of patients and personnel at the time of their arrival and departure from hospitals and radiology establishments, are considered mandatory. M. Palacios Montesinos further explained that working hours in many places were modified and “home office (teleradiology)” was implemented, reducing the risk, the number of working hours and coexistence in small spaces, thus isolating doctors, especially those over 60 years of age and those with comorbidities such as obesity, diabetes, hypertension and lung disease. Specific care hours were generated for patients with suspected COVID-19 and in hospitals with more than one CT scanner, one was designated for their diagnosis. Specialized isolation areas were designated in each hospital for COVID-19 positive patients. M. Palacios Montesinos concluded that health care system in Mexico is insufficiently equipped to provide the level of medical care that would guarantee the minimum necessary care for these patients and even less so in severe cases and that, as a result, the infections and death figures reported by the government do not come close to reality for a country with approximately 120 million inhabitants.

M. Arrieta, reporting on behalf of the **Colombian Association of Radiology (ACR)**, stated that the ACR designed and developed different education and support strategies for radiologists and the medical community in the scientific and trade field. To this end, in March, groups of experts were formed to prepare guidelines and recommendations addressed to diagnostic imaging services, radiologists, radiographers, and care and administrative personnel. Similarly, the ACR Scientific Committee organized virtual education activities to inform and train the country's radiologists about the new virus. Furthermore, between March and June the ACR published 10 different guidelines and participated in the development of a “Colombian Consensus on Care, Diagnosis and Management of SARS-CoV-2/COVID-19 Infection in Health Care Facilities” [2], a document that has been the basis of government policies for the management of the pandemic. A special section on COVID-19 on the ACR website allows consulting and downloading these and other documents of interest. The ACR has also actively participated in various meetings convened by Colombian medical organizations to analyze the situation generated by the pandemic and define official positions of the union. Thanks to the ACR's efforts with the Ministry of Health and the National Institute of Health, it was possible to include diagnostic imaging (chest studies) in the COVID-19 national care guide. Reporting on the challenges facing Colombian radiologist, M. Arrieta pointed out that many health professionals on the front line of care do not have adequate personal protection equipment and others, working in outpatient centers, have been fired from their jobs. For this reason, the ACR has implemented an exclusive service for their members, offering them information and legal advice pertaining to labour guarantee demands towards their employers and insurance companies. Finally, M. Arrieta stated that, like many other scientific societies in the world, the ACR was forced to cancel or postpone all of its academic activities scheduled for 2020, including the Colombian Congress of Radiology. In response to this situation, in 2020 the ACR has expanded their virtual education program, with monthly virtual courses, weekly webinars and online academic sessions on different areas of the specialty. In April, the ACR carried out live broadcast of 2 cycles of virtual academic sessions on COVID-19 (12 lectures in total), with the participation of specialists from different medical disciplines and a special emphasis on diagnostic imaging. Each of these sessions registered an attendance of over 700 participants from 25 countries.

H. Díaz Lazo provided a report on behalf of the **Peruvian Society of Radiology (SOCPR)**. Reporting on the effect the virus has had on the entire country and the capital Lima, H. Díaz Lazo explained that in order to prevent the spread of COVID-19, the Government has decreed that the population must remain in home isolation. This measure, however, proved not to be particularly effective. As a result, the Peruvian health system is on the verge of collapse, with hospitals being oversaturated and in some centers the lack of equipment and protective materials is leading to a daily increase in the number of positive cases. Given this situation, the SOCPR has found it necessary to emphasize the various recommendations in order to strengthen the health system in response to COVID-19. To that end, reaffirming several recommendations given by The World Health, the SOCPR has provided protective equipment and helmets with fascial protection to all associated radiologists and physician residents throughout the country. Additionally, to optimize social protection and frontline workers’ health, the SOCPR has encourage health training related to radiology. H. Díaz Lazo explained that in the last weeks the SOCPR has organized five webinars, with the participation of national and international experts. Finally, the SOCPR has published a special edition of the Peruvian Radiology Magazine “Radiología del COVID-19” with various highly search topics that are also of public interest.

A. Sarmet Santos, on behalf of the **Brazilian College of Radiology (CBR)**, reported on the situation in Brazil. He explained that the CBR, through its Executive Board, its Scientific Departments, and its Commissions, has been constantly active and participated in numerous meetings with other Brazilian and international institutions, pointing out CBR’s participation in the “Covid-19 Collaborative Force—Brazil”, a group that provide advice to the National Health Surveillance Agency (Anvisa), for the preparation of Technical Notes referring to COVID-19, which have the force of law in Brazil and must be followed by all Health Institutions, public and private. A. Sarmet Santos further mentioned that the first “CBR Studio” of 2020 was held in February as a live webinar, with the focus on COVID-19 and that in March, hundreds of radiologists attended a lecture by Prof. Dr. Dante Escuissato on “Viral Pneumonias: Radiology-Pathology and Coronavirus / COVID-19 Correlation”, through the CBR virtual platform. In April, the CBR launched the Virtual Learning Environment, a new learning space for the Brazilian radiologists where they can receive constant updates. The classes and lectures of the Brazilian Congresses of 2018 and 2019 were also made available on the platform. Due to the social isolation measures imposed by the Brazilian authorities, CBR provided full and free access to the content available on the Platform, initially for a period of 30 days and later extended until July 31, 2020, in order to ensure updating and recycling of knowledge among Brazilian radiologists. Additionally, A. Sarmet Santos pointed out that, already in April, the CBR mobilized suppliers in the business segment, aiming to renegotiate contracts or make debts and payments for inputs flexible, and ensure maintenance contracts and purchases of new equipment for Image Diagnosis clinics. The CBR’s goal in this regard was to economically protect the sector that was already suffering from the impact and losses in the face of the COVID-19 crisis. In addition, in order to gather communications, recommendations, and alerts, CBR developed a dedicated space for content and publications related to COVID-19 on the CBR website. The main purpose of this virtual space is that radiologists and diagnostic imaging professionals, medical colleagues from other specialties, professionals from different areas of healthcare in general, journalists and opinion makers, and, mainly, the population, have continuous and free access to relevant information. In addition to the CBR recommendations, the CBR uses this space to gather guidelines from other medical societies as well. Finally, A. Sarmet Santos According explained that, similar to other medical societies, the Brazilian Congress of Radiology 2020, which would be held in the city of Rio de Janeiro in October 2020, will be 100% virtual.

M. Brandão da Costa, reporting on behalf of the **Radiological and Diagnostic Imaging Society of Sao Paulo (SPR)** pointed out the social and economic inequalities that are present in Brazil and that have contributed to challenges experienced in the fight against COVID-19. When reporting on the effects of the pandemic on radiologists in Sao Paulo, M. Brandão da Costa explained that the number of routine examinations dropped down considerably and that only emergency or seriously ill patients were examined. From March to June, most radiologists worked from home, in reduced time. The residency programmes were also heavily affected as teaching depends on the variety of conducted examinations (CT, MRI, US) and areas (Neuro, Abdominal, Breast, Chest), but most centers were focused exclusively on COVID-19 diagnosis. Realizing the importance of new information when fighting COVID-19 and taking into account the numerous social and economic difficulties Brazil is experiencing, the SPR took action and supported radiologists fighting COVID-19 through several projects. Jornada Paulista de Radiologia (JPR), which is considered the largest Diagnostic Imaging meeting in Latin America and the fourth largest in the world, was organized as a virtual meeting with 23 Live Sessions, 6 Plenary Sessions, and more than 170 recordings that are going to be available to SPR members for free. In order to increase the access to the materials shared at the JPR 2020, the SPR has offered free membership to everyone who is an active member of any of the SPR partner radiological societies in Latin America. The SPR also created a dedicated online area that offers reliable information on COVID-19, such as articles, guidelines, recommendations, and webinars. M. Brandão da Costa particularly emphasized that for the JPR Lives and Study Groups Meetings, the SPR chose topics related to COVID-19 that can thus be made freely available in this dedicated online area. Elsevier offered 800 STATdx® free licenses to SPR, that are to be distributed among SPR members and Radiology Departments and Clinics all over Brazil, which had subscribed for the SPR Online Radiology Course for Residences before. Finally, M. Brandão da Costa stated that in April and May, when many residents and radiologists were working exclusively from home due to social distancing policies, SPR offered free credits for the SPR Video Library to SPR active Members and access to the SPR Video Library during this period was greatly increased.

H. Carrete Junior, representing the **Interamerican College of Radiology (CIR)**, explained that from the onset of the global pandemic the CIR worked on the organization and distribution of statements and guidance materials created by different national radiological societies. Additionally, several national societies used the resources of CIR to promote different webinars focused on the COVID-19 pandemic, which were thus widely promoted among Latin American radiologists leading to hundreds of members participating in these events. In recent months, discussion forums and several online lectures focusing on the COVID-19 pandemic have been organized and widely disseminated through CIR channels. Some previously scheduled scientific events had to be reorganized as virtual events exclusively, and such events were offered online to a larger community in Latin America, in addition to pandemic-related topics being included in the scientific program. H. Carrete Junior concluded that through its work the CIR has created a huge network of collaboration, and radiology once again showed its well-structured organization, having recognized since the beginning of the pandemic its important role in facing this outbreak.

## The situation in Egypt

T. El-Diasty, on behalf of the **Egyptian Society of Radiology and Nuclear Medicine (ESRNM)**, submitted a report on the current situation in Egypt. He stated that in resource-constrained environments, as is the situation in many governorates in Egypt, the decision to image patients who are COVID-19 positive or suspected is based on how the imaging will impact patient care. Imaging is indicated for patients with suspected COVID-19 who present moderate to severe clinical features and a high pre-test probability of disease. However, the use of CT as a primary screening tool is discouraged, because these studies tend to suffer from selection bias. From its side, the ESRNM has highlighted the different guidelines, publications, and opinions about this issue with T. El-Diasty explaining that chest radiography (CXR) is the first-line imaging examination in patients suspected of having COVID-19 infection and CT being necessary whenever indicated. T. El-Diasty further explained that the real-time reverse transcriptase polymerase chain reaction (RT-PCR) test for COVID-19 is reported to have high specificity but sensitivity has been reported to be as low as 60%–70% and therefore, excluding a diagnosis of COVID-19 requires multiple negative tests, with kits being in short supply or unavailable in some regions of Egypt. In response to reports of lung abnormalities on CT predating conversion to positive RT-PCR, Egyptian authorities initially broadened the official definition of infection to include patients with typical findings at CT, even with a first negative RT-PCR result. This broader definition has resulted in a higher number of presumptive cases of COVID-19 and an increasing role for CT in diagnosis. However, the presence of mild or no CT findings in many early cases of infection reflects the difficulties of early detection.

## The situation in the United Arab Emirates

Dr. AlBastaki, on behalf of the **Radiology Society of the Emirates (RSE)** explained that, due to the close contact with the countries that experienced the pandemic early, such as China and Iran, the United Arab Emirate managed to prepare early for the pandemic. As such, the national guidelines were formed and were updated four times. Radiologists across the country have adopted different approaches when dealing with the disease. In this regard, Dr. AlBastaki stated that some radiologists preferred to follow early papers that have been published from China as well as a report from Italy indicating the necessity of performing CT in all COVID patients, while others have decided to follow the ACR and the RCR guidelines of performing CTs only when it is clinically indicated. A chest x-ray is being performed routinely for all patients, with portable x-ray labs being available in the hospitals, quarantine centers, and field hospitals. Dr. AlBastaki further reported that the safety of the radiology staff was extremely important, particularly on the front line with radiographers performing mobile chest-x-ray and radiologists doing intervention procedure. From its side, the RSE has organized two dedicated webinars in cooperation with Basildon University Hospital in the UK, to show the best international experiences in facing COVID-19. The webinars were available for radiologists and radiographers and were accredited by the royal college of radiologists in London. Finally, Dr. AlBastaki stated that staff clinics were organized where staff could express their concerns and throat swab tests are being performed if COVID-19 is suspected, with periodic screenings being performed as well.

## The situation in India

D. Patkar, on behalf of the **Indian Radiological and Imaging Association (IRIA)**, reported that, in order to facilitate smooth functioning of the radiology departments, they are implementing a strategic approached named “AMMO”: Analyse, Minimise, Mobilise, Organise. Explaining individuals points of the strategy, D. Patkar reported that “Analysis” includes patient’s COVID-19 status, including triage at multiple levels as well as the analysis of need for imaging, and stated that during the initial months of the pandemic non-emergency scans were being postponed, but that now routine imaging is resuming. D. Patkar further explained that one of the main aims is to minimize exposure time, with SOPs being implemented, numbers optimized, department workflow restructured, portable imaging made available, and rotational shifts introduced. Reporting on the “Mobilise” aspect of the strategy, D. Patkar reported that portable radiography and ultrasound systems have been allotted for COVID-19 scans, provisions were made for radiologists to work from home and teleradiology services and various AI based software are also being utilized. Approximately 25% of the radiologists have been re-routed to COVID-19 services, as per the need of the institution. Finally, explaining the “Organise” aspect of the strategy, D. Patkar stated that dedicated COVID-19 corridors are being created for mobilization of positive/suspected patients within hospital premises, appointments are being scheduled with adequate time spacing, and deep cleaning of equipment is done after every patient, with a down time of approximately 1 h.

## The situation in Thailand

W. Tanomkiat, reporting on behalf of the **Royal College of Radiologists of Thailand (RCRT)** and the **Radiological Society of Thailand (RST),** stated that disease control laws were passed during the early stage of spreading, by the government. These laws were strictly applied and observed and communities in each district were reduced to “Home” and “Hospital” with most community areas other than Home and Hospital being almost entirely closed. Furthermore, hospital areas were clearly divided into the front line and the clean zone and prevention of nosocomial infection to either in-patients or health care providers was regarded as crucial. As a result, nosocomial infection occurred only in 2 out of more than 1,300 hospitals. Mobile network was well developed, covering all parts of the country and the high adoption of Internet allowed e-commerce, e-learning, e-meeting, e-entertainment, telemedicine, and telepharmacy to substitute these activities which were limited in the physical world. W. Tanomkiat also explained that public education on the COVID-19, its nature and route of spreading was thoroughly disseminated through various media including television, radio, and electronic media by the government authorities. From its side, the RCRT recommended that radiologic images should not be used in detection or confirmation of the COVID-19 pneumonia because of certain false positive and negative rates. Images were requested by the official caring physician team of the hospital and performed under recommended protocols. Transferring patients to the isolated radiologic department was operated by a central team. Types of appropriate protective equipment and standard of operation for each study were clearly provided. Finally, W. Tanomkiat concluded that there are no radiologists or radiological technologists infected with COVID-19 in Thailand.

## The situation in China

Z. Y. Jin, on behalf of the **Chinese Society of Radiology (CSR),** reported on the situation in China. He stated that China has taken multiple measures to combat COVID-19, benefitting from the valuable experience during the Manchurian plague from the previous century. Based on that experience the Chinese government and public health department promptly established an emergency response scheme in the very early stage of COVID-19, emphasizing early detection, early reporting, early isolation, and early treatment. A unified and effective commanding system, a series of law-based and science-driven strategies, the ‘4-Early’ measure, the coordinated deployment of resources, the rapid improvement in treatment capacity, the application of high-tech measures, and international exchange and cooperation substantially mitigated the spread of COVID-19 in China. Reporting on the activities of the CSR, Z. Y. Jin explained that in February 2020, the Chinese Society of Radiology organized members from different parts of China to help improve medical quality and security when diagnosing COVID-19. Experts in cardiothoracic branch published an expert recommendation in the Chinese Journal of Radiology entitled “The radiological diagnosis of novel coronavirus pneumonia: expert recommendation from the Chinese Society of Radiology (First edition)” [3]. Another message that the CSR sent is that chest CT findings can be not parallel to nucleic acid detection. The Chinese Society of Radiology strongly recommend that the physicians and radiologists should carefully consider the negative result from chest CT and nucleic acid test during the clinical procedure. Reporting on the Chinese experience in hospital environment preparedness for COVID-19, Z. Y. Jin stated that hospitals in China including Peking Union Medical College Hospital (PUMCH) have heightened the security on the whole campuses, as screening is the first and fundamental step of the flow and that PUMCH urgently set up a series of separate cabinets with air conditioners for nasopharyngeal swab collection. The CSR therefore appeals to the global community for the manufacture, equipment, and utilization of integrated cabinets. Furthermore, the CSR emphasized that environmental services staff members need to be specially trained for professional cleaning of potentially contaminated surfaces after each high-risk patient contact. Radiology departments should contact their equipment vendors to find the safest disinfectant for each piece of equipment in use. Additionally, the CSR encourages the radiology departments to collaborate with the IT departments to construct a remote imaging system helping radiologists work at home. Moreover, a medical application on both iOS and Android smart phones can help patients receive their image reports. Z. Y. Jin also emphasized the importance of collaboration between the radiology community and local government in order to raise public awareness in disease control, stating that the CSR has published a free handbook on COVID-19 for the public. Finally, Z. Y. Jin commended the strong international collaboration between different radiological societies.

## The situation in Korea and Japan

W. Lee submitted a report on behalf of the **Korean Society of Radiology (KSR).** He emphasized the importance of scientific reports, mentioning that a Korean chest radiologist promptly published initial experience in the Korean Journal of Radiology [4]. W. Lee further explained that the KSR and the Korean Society of Thoracic Radiology (KSTR) published “KSR/KSTR Guidelines for the use of Diagnostic imaging for COVID-19” [5], the recommendations regarding the use of diagnostic imaging for COVID-19 in various clinical scenarios. The published Guidelines focused on the role of imaging studies (chest radiographs, chest CT) as screening tests for COVID-19, the use of diagnostic imaging for COVID-19 patients in community treatment centers for isolation and imaging studies for admitted patients with COVID-19. Korean Society of Thoracic Radiology (KSTR) recently established and published Korean Imaging Cohort of COVID-19: Potential Role in Education and Research [6].

S. Aoki, on behalf of the **Japan Radiological Society (JRS)**, reported that due to the geographic proximity to China, Japan was one of the first countries to be affected by the outbreak. It was quickly realized that the basic supplies were lacking, including personal protective equipment (PPE) and the tests for diagnosis (i.e. reverse transcription polymerase chain reaction: RT-PCR), thus having to rely heavily on contact-tracing (AKA “cluster busters”). S. Aoki further reported that, in addition to the initial outbreak, Japan was faced with the cruise ship Diamond Princess in early February, which was the largest cluster outside of China. However, one of the first imaging studies [7] was performed exactly on this group of patients, where quarantined PCR positive patients underwent screening CT scans and the results of this study have shown that the sensitivity of CT in detecting the lung diseases in asymptomatic patients was not high with only half of those who were confirmed positive for COVID-19 had CT evidence of viral pneumonia. Commenting on the use of CT, S. Aoki reported that the JRS made a few announcements on their web sites stating that “CT is not for screening” [8, 9]. Another project mentioned by S. Aoki was the national survey aimed at capturing the trend of viral pneumonia as depicted by CT [10]. The survey was carried out through the untiring volunteer work of the radiologists at the frontline. They were asked to click through a web-based survey to keep record of the cases they encountered. All suspected cases were included, both with and without PCR diagnosis of COVID-19. Of note is that the incidence of “pneumonia of unknown aetiology” had a similar trend to the number of proven cases. This suggests that there are clinically undiagnosed cases. Finally, S. Aoki stated that this pandemic demands further international collaboration.

## The situation in the Asia-Oceania region

D. Varma, on behalf of the **Asian Oceanian Society of Radiology (AOSR)**, reported on the current situation, activities and measures undertaken by radiologists in Asian Oceanian region. In Australia, the hospitals went into overdrive increasing ICU bed capacity, ceasing elective surgery separating areas in Emergency Departments for suspected COVID-19 cases. PPE was used widely in managing these patients. Some radiology departments managed to separate imaging and interventional facilities to look after COVID-19 suspect or confirmed cases. In Hong Kong, The Hong Kong College of Radiologists (HKCR) aligned radiologists from all Radiology Training Centers in Hospital Authority (HA). Departments engaged occupational therapists to design an ‘anti-splashing screen’ to protect the staff during ultrasound scan aiming to conserve PPE. Japan Radiological Society (JRS) has started surveillance on diagnostic imaging of virus pneumonia in order to grasp the novel coronavirus clusters and the spread of the virus infection in Japan. Malaysia hospitalized all COVID-19 positive individuals in designated hospitals. Private hospitals were on standby to receive non-COVID-19 Ministry of Health (MOH) patients. Radiology departments took steps to be “COVID-ready”. Regularly updated MOH guidelines on COVID-19 management were widely circulated. In Korea, the Korean Society of Radiology and the Korean Society of Thoracic Radiology have prepared recommendations for the use of diagnostic imaging for COVID-19 in various clinical scenarios. On March 23, 2020, the Royal College of Radiologists of Thailand and Radiological Society of Thailand established “Guidelines for radiology practice during pandemic of COVID 19 for Thai radiological healthcare professionals”. The Thailand Ministry of Digital Economy and Society and a private firm are conducting limited use and research using artificial intelligence (AI) to detect COVID-19 pathology on CT chest. These products are however not yet commercially available. In Taiwan, in 2004 following the SARS (severe acute respiratory syndrome) outbreak, the government established the National Health Command Centre (NHCC) overseen by Ministry of Health and Welfare. The NHCC has been crucial in gathering early data on the COVID-19 outbreaks in China and other parts of Asia. Taiwanese model of epidemic preparedness from previous experiences has proven of great value in containing the spread of COVID-19.

## The situation in Australia and New Zealand

L. Lawler reported on behalf of the **Royal Australian and New Zealand College of Radiologists (RANZCR)**. The RANZCR took measures to reduce risks and ensure the safety of its members and their patients and at the beginning of March 2020 formed a COVID-19 Taskforce. The Taskforce, made up of senior clinicians including representatives from the Faculty of Clinical Radiology and Faculty of Radiation Oncology Councils, Chief Censors from each faculty, Chair of the Safety and Quality Committee, a representative from the Board, RANZCR CEO, and a media and communications staff member, was responsible for coordinating a whole-of-College response to the COVID-19 outbreak that effectively mitigated the risks to clinical radiologists, their patients, the College and College staff. With oversight from the COVID-19 Taskforce, RANZCR developed several position statements and guidance documents to support members. RANZCR also worked closely with governments and other stakeholders to advocate for appropriate access to personal protective equipment (PPE), and monitored emerging literature from around the world to share with members as educational resources. These multidisciplinary resources included key links to educational material on imaging findings and information on how to prepare clinical radiology departments and practices to cope with the challenges presented by COVID-19. L. Lawler further explained that RANZCR advocated strongly to governments to provide reassurances that vulnerable clinical radiology practices could remain viable during the pandemic period, as the pandemic rapidly and severely affected private and community-based radiology services throughout Australia and New Zealand, to the extent that many diagnostic and interventional practices faced real concerns over their long-term viability. Finally, L. Lawler concluded that, concerningly, deferred referrals to radiology due to COVID-19 were likely to significantly extend the waiting times for patients and would put increased pressure on radiology services into the future due to the backlog.

## The situation in Europe

B. Brkljačić reported on behalf of the **European Society of Radiology (ESR)**. He explained that the ESR was fast to react to the effects of the pandemic and developed a strategic approach aimed at facilitating the fight against the virus and supporting radiologists in their work with COVID-19 patients and advocating the importance of ensuring continuity of care for all other patients. Already in the first days of the pandemic, the ESR established a dedicated resource hub on the ESR website with important resources for radiologists. The resource hub contains information on guidelines, publications, research, and support measures with the data being continuously updated. Furthermore, the European Institute for Biomedical Imaging Research (EIBIR) compiled a preliminary list of open access COVID-19 datasets that can be used for teaching, training, and research. The list is being continually updated with new databases and registries, as radiologists around the world continue making progress in fighting the pandemic. Through ESR Connect, the Society’s video platform, ESR provided a medium for leading scientists to present latest findings related to the ongoing pandemic. Series of “ESR Connect Special Reports—Radiology Fighting COVID-19” offered first-hand reports from European experts sharing their countries’ experience in diagnosing and treating COVID-19 patients. In addition to being available on ESR Connect, all recent COVID-19 streaming events have also been published on ESR YouTube channel to guarantee easy sharing and simple accessibility to the latest knowledge and findings. In order to provide further support to radiologists around the world fighting this emergency, the ESR decided to make its online platforms ESR Connect and Education on Demand freely available for a limited period. With these services, a wealth of video content, recorded lectures, literature, and educational courses were made readily available for everyone completely free of charge. Furthermore, as increased connectivity and information exchange have proven to be vital when dealing with a global pandemic, the ESR intensified its social media presence, striving to foster further discussion among imaging professionals through various online channels, such as the dedicated ESR Twitter discussion thread. At the same time, the ESR released statements aimed at politicians in the European Union, emphasizing the importance of medical imaging in the fight against COVID-19 and calling for healthcare systems to implement multidisciplinary and integrated solutions to offer the right diagnostic and treatment options to patients. The Society’s Annual Meeting, ECR 2020, was also affected by restrictions imposed by the national governments and was successfully organized as a virtual event. Carefully designed scientific programme at ECR 2020 included special sessions dedicated to the fight against COVID-19 with foremost European experts offering their knowledge and experience. The 2020 International Day of Radiology (IDoR) is dedicated to all imaging professionals and their essential role in fighting the COVID-19 pandemic making an indispensable contribution to the diagnosis and treatment of COVID-19 patients. With the IDoR 2020 motto ‘Radiologists and radiographers supporting patients during COVID-19’ the ESR strives to call attention to the critical role that medical imaging has played during the current crisis. Finally, B. Brkljačić explained that, despite the difficulty of the current situation, the societies must look to the future and analyze the long-term consequences that the COVID-19 pandemic will have on different aspects of radiology. An important issue in this regard is the education and training of future radiologists that has been disrupted by the effects of the pandemic. That is why the ESR has developed a survey addressed to young radiologists across Europe that aims to examine what were the effects of the pandemic on their education and training, in order to be able to mitigate the pandemic’s negative consequences on future generations.

## Position of the International Society of Radiology (ISR)

L. Donoso, on behalf of the **International Society of Radiology (ISR)**, reported that, following the outbreak of the COVID-19 pandemic, the ISR launched a number of initiatives, which showed the importance of ISR collaboration with the WHO and the International Atomic Energy Agency (IAEA). The ISR set up a collection of freely accessible resources on its website, including guidelines provided by member societies from all over the world as well as open source publications and websites on Covid-19 and WHO advice including ISR input. Furthermore, to support countries in their response to the COVID-19 pandemic, WHO has developed a Rapid Advice Guide on the use of chest imaging in COVID-19 [11] which provides health care professionals with recommendations on the appropriate use of different imaging modalities in COVID-19 cases to support diagnosis and patient management decisions, considering different settings around the world. These recommendations were developed by a multidisciplinary group of experts and relevant stakeholders from all over the world in liaison with the Core Group, both under the chairmanship of Prof. Guy Frija, who is also Co-Chair of the ISR Quality and Safety Alliance and Chair of the ESR EuroSafe Imaging Steering Committee. L. Donoso emphasized that the ISR provided technical support for data collection on imaging practices in COVID-19 management to inform this guide, which included the development and dissemination of a survey, conducted jointly with the ESR, which in turn showed considerable variations in imaging practices related to COVID-19 across the world. A report^12^ on the survey results was subsequently published. Through the ESR, ISR also provided virtual meeting assistance and rapporteur services. L. Donoso concluded by stating that, due to the novelty of the coronavirus disease, evidence and data are continuously emerging which is why the WHO will closely monitor the development and consider an update of the rapid advice guide within the next six months.

## Conclusion

The global pandemic of COVID-19 has had an unprecedented impact on the modern world and is unlike anything experienced in generations. There exists not a single aspect of people’s daily lives that has not been, directly or indirectly, affected by the pandemic. While the scientists around the world are in a race against time to create a suitable vaccine that could help defeat the virus, the medical staff is doing their best to mitigate the consequences that the virus is having on human health and to save as many lives as possible. However, hospitals around the world are experiencing over saturation, and lack of staff and suitable equipment is making an already difficult fight even more complicated. Radiologists play an especially important role in the global efforts against the pandemic, and radiological societies around the world are doing their best to support not only the medical staff but also the governments and the general public in the joint struggle against this vicious enemy.

The radiological societies in North America are joining efforts with other societies and are organizing dedicated workgroups and task forces that will manage to tackle current problems in a focused yet flexible way. At the same time, they are creating knowledge repositories and offering much needed guidelines, statements and recommendations addressed to the governments as well as the general public. Only through the joint efforts of the radiological societies, the government, industry, and stakeholders can proper information and advice be provided which will lead to minimizing the spread of COVID-19.

In Latin America, the societies are being faced with a critical lack of protective equipment and materials while at the same time experiencing a surge in the numbers of confirmed cases, with many healthcare systems being on the verge of collapse. The societies are offering much needed guidelines and recommendations on how to deal with the crisis and constant online educational activities are being provided. Latin American radiologists are forced to adapt and to find innovative ways of dealing with the pandemic while trying to protect the patients’ health.

Many areas of Egypt are also being faced with a lack of resources, materials, and equipment, however the ESRNM is relentless in highlighted the different guidelines, publications, and opinions that will help radiologist fight this pandemic. In the United Arab Emirates, the healthcare system was relatively well-prepared to deal with the pandemic and radiologists in the country are carefully examining developments and research findings coming from other countries. In India, a specific strategic approach, named “A.M.M.O.” has been developed, aimed at analyzing the patients’ status, minimizing exposure risk, mobilizing the available radiologists and organizing the workflow in the hospitals in a way that will protect the patients. In Thailand, the online aspects of different activities are being implemented and radiologists are discouraging an over-extensive use of imaging when diagnosing COVID-19. China, as a country that was the first to experience the deadly consequences of the COVID-19 pandemic, is using this experience to share the knowledge and offer guidelines and recommendation to other societies across the world. Research papers from China have served as stable source of information in the fight against the virus and the CSR is offering different recommendations based on the Chinese experience, such as the one to implement the use of integrated cabinets when dealing with the virus. In Korea, the KSR is emphasizing the importance of scientific research and in Japan, the JSR is continuously offering recommendations based on latest surveys and findings.

When it comes to the Asian Oceanic region, individual societies are mobilizing all available resources to fight the virus and are trying to minimize its spread. Accordingly, the RANZCR formed a COVID-19 Taskforce that oversaw the development of several position statements and guidance documents to support the RANZCR members. At the same time the RANZCR is working closely with the government to provide reassurances that vulnerable clinical radiology practices could remain viable during the pandemic period, as the pandemic rapidly and severely affected private and community-based radiology services throughout Australia and New Zealand.

In Europe, the ESR has developed a focused strategic approach that emphasizes facilitated access to the latest knowledge and enables experience sharing among radiologists. At the same time, the ESR looks to the future and strives to analyze what the long-term consequences of COVID-19 on radiology will be.

The ISR is working closely with WHO and the IAEA and is providing healthcare professionals with recommendations on the appropriate use of different imaging modalities in COVID-19 cases to support diagnosis and patient management decisions.

Radiologists around the world are being faced with one of the deadliest threats the modern world has experienced. The constant flow of knowledge and sharing of best practices, recommendations, and guidelines as well as closer than ever collaboration between different radiological societies are paramount in ensuring that this unprecedented threat is overcome.

## Data Availability

Not applicable.

## Abbreviations

AAPM: American Association of Physicists in Medicine
ACR: American College of Radiology
ACR: Colombian Association of Radiology
AI: Artificial intelligence
Anvisa: National Health Surveillance Agency
AOSR: Asian Oceanian Society of Radiology
APPs: Advanced Practice Providers
CAR: Canadian Association of Radiologists
CBR: Brazilian College of Radiology
CIR: Inter-American College of Radiology
CSR: Chinese Society of Radiology
CXR: Chest radiography
ECR: European Congress of Radiology
EIBIR: European Institute for Biomedical Imaging Research
ESR: European Society of Radiology
ESRNM: Egyptian Society of Radiology and Nuclear Medicine
FMRI: Mexican Federation of Radiology and Imaging
HA: Hospital Authority
HKCR: Hong Kong College of Radiologists
IAEA: International Atomic Energy Agency
IDoR: International Day of Radiology
IRIA: Indian Radiological and Imaging Association
ISR: International Society of Radiology
JPR: Jornada Paulista de Radiologia
JRS: Japan Radiological Society
KSR: Korean Society of Radiology
KSTR: Korean Society of Thoracic Radiology
NHCC: National Health Command Centre
NIBIB: National Institute of Biomedical Imaging and Bioengineering
NIH: National Institute of Health
PPE: Protective Personal Equipment
PUMCH: Peking Union Medical College Hospital
SARS: Severe acute respiratory syndrome
SMRI: Mexican Society of Radiology and Imaging
SOCPR: Peruvian Society of Radiology
SPR: Radiological and Diagnostic Imaging Society of Sao Paulo
RSE: Radiological Society of the Emirates
RSNA: Radiological Society of North America
RST: Radiological Society of Thailand
RANZCR: Royal Australian and New Zealand College of Radiologists
RT-PCR: Reverse transcriptase polymerase chain reaction
RCRT: Royal College of Radiologists of Thailand
